# Semicircular canal shape diversity among modern lepidosaurs: life habit, size, allometry

**DOI:** 10.1186/s12862-023-02113-1

**Published:** 2023-04-12

**Authors:** Ashley E. Latimer, Emma Sherratt, Timothée Bonnet, Torsten M. Scheyer

**Affiliations:** 1 Department of Palaeontology, University of Zurich, Karl Schmid-Strasse 4, 8006 Zurich, Switzerland; 2 School of Biological Sciences, The University of Adelaide, Adelaide, SA 5005 Australia; 3 Research School of Biology, Australian National University, Canberra, ACT 0200 Australia

**Keywords:** Squamata, Lepidosauria, Inner ear, Semicircular canal shape, Geometric morphometrics

## Abstract

**Background:**

The shape of the semicircular canals of the inner ear of living squamate reptiles has been used to infer phylogenetic relationships, body size, and life habits. Often these inferences are made without controlling for the effects of the other ones. Here we examine the semicircular canals of 94 species of extant limbed lepidosaurs using three-dimensional landmark-based geometric morphometrics, and analyze them in phylogenetic context to evaluate the relative contributions of life habit, size, and phylogeny on canal shape.

**Results:**

Life habit is not a strong predictor of semicircular canal shape across this broad sample. Instead, phylogeny plays a major role in predicting shape, with strong phylogenetic signal in shape as well as size. Allometry has a limited role in canal shape, but inner ear size and body mass are strongly correlated.

**Conclusions:**

Our wide sampling across limbed squamates suggests that semicircular canal shape and size are predominantly a factor of phylogenetic relatedness. Given the small proportion of variance in semicircular canal shape explained by life habit, it is unlikely that unknown life habit could be deduced from semicircular canal shape alone. Overall, semicircular canal size is a good estimator of body length and even better for body mass in limbed squamates. Semiaquatic taxa tend to be larger and heavier than non-aquatic taxa, but once body size and phylogeny are accounted for, they are hard to distinguish from their non-aquatic relatives based on bony labyrinth shape and morphology.

**Supplementary Information:**

The online version contains supplementary material available at 10.1186/s12862-023-02113-1.

## Background

All animals, whether they run, swim, fly, crawl, or burrow, must be able to orient themselves in a three-dimensional environment. In vertebrates this ability is mediated by the inner ear, particularly the semicircular ducts, which are vital to balance, orientation, head position, and gaze stabilization [1, 2]. Unlike vision, and like touch, this sensory system is never wholly lost in any group of animals. This means every vertebrate has this feature, and it provides a powerful near-unique means of comparison among all vertebrate taxa. Indeed, although some features (cross-sectional areas of the slender parts of the ducts and cupula anatomy) are not preserved, the general shape of the soft tissue semicircular ducts is preserved by the bony semicircular canals. The canals are easily imaged in well preserved skulls in living animals or extinct, bone or fossil, providing the comparison in any vertebrate where a skull remains. The semicircular canals have proven a rich source of data for morphological comparison and inferring ecology, posture, locomotion, and phylogeny [3–7]. The semicircular canals of mammals are the most studied (e.g. [8–13]), recently bolstered by a surge in research of reptiles and birds [5, 14–22], amphibians [23, 24] and fishes [25].

Although the lion’s share of work has been done on mammals, recent work on reptiles and amphibians upholds similar relationships between semicircular canal morphology and ecology. Semicircular canal morphology has been tentatively linked with life habits including fossoriality and burrowing [5, 17, 23], gliding [15], as well as to the degree of adaptation to aquatic [16, 26], and arboreal habitats [18, 27]. Unique locomotion, also, has been tentatively associated with unique semicircular canal morphology, e.g. tail-assisted walking used by dwarf chameleons is associated with short and bulbous semicircular canals [27]. Following these propositions, comparative anatomy of the semicircular canals of extant species has been used to infer life habit of extinct taxa [5, 16, 17, 26, 28]. But, for distantly related clades, do the shapes of the semicircular canals become more similar with similar life history and despite phylogeny? Following that, can the information in the shape of the semicircular canals be informative enough to confidently reconstruct the life habit in extinct members of these groups? Mixed results on restricted clades suggest not [5, 18], but this relationship has not been tested for total group squamates.

Unlike mammals, in reptiles the bones comprising the semicircular canals also form the side wall of the braincase, and coevolution between the seemingly isolated semicircular canals and head proportions influences canal morphology (e.g., [29]). Cranial bones may even remain un-sutured in some reptiles until either adulthood or a large body size is achieved, or remain unconnected in death [30, 31], leaving the potential for shape change in the semicircular canal system through life. Ontogenetic change in skull shape between clades, and correlations between environmental factors (e.g., rainfall) and head shape have been investigated for only a few clades [31, 32]. However, relationships between head shape and canal morphology have been identified within genera [18]. Moreover, these relationships depend on head size [19], and ellipticity of canals [33]. Whether the shape of the inner ear covaries with size across clades (e.g., evolutionary allometry [34]), however, has not been tested.

We test whether semicircular canal shape is correlated to life habit, size and shape and body mass across clades of squamate reptiles. Specifically, we investigate the relative contribution of phylogenetic history, four life habit groups (semiaquatic, arboreal, fossorial, and terrestrial), and size on semicircular canal shape across living squamates with limbs, thus excluding limbless lizards and snakes, which have different effects of size on ecology [35] and have been studied elsewhere [5].

## Methods

### Specimens and CT scans

The endocasts of the bony labyrinth of 93 species of limbed squamates and *Sphenodon punctatus* were digitally sectioned and extracted from X-ray micro computed tomographs in VG StudioMax 2.2 (Additional file 1: Fig. S1). Tomographs are from three sources based mainly on specimen availability at the time of data assembly: (1) *Amevia amevia*, *Iguana iguana*, *Saara hardwickii*, *Varanus griseus*, *V. niloticus*, *V. rudicollis*, *V. salvator* were scanned by A.L. at the University of Zurich; (2) Australian varanids (*V. brevicauda*, *V. bushi*, *V. caudolineatus*, *V. eremius*, *V. giganteus*, *V. gilleni*, *V. glauerti*, *V. mitchelli*, *V. panoptes*, *V. rosenbergi*, *V. scalaris*, *V. storri*, *V. tristis*) were scanned at the Australian National University for J. Scott Keogh; (3) the remainder were sourced from scans in the repository Digimorph, and were originally part of the Deep Scaly Project [36]. In our sample, the left semicircular canals were extracted. Where the left vestibular system was not available, we mirrored the right one, since left–right asymmetry is negligible herein.

### Landmarks

**Fig. 1:**
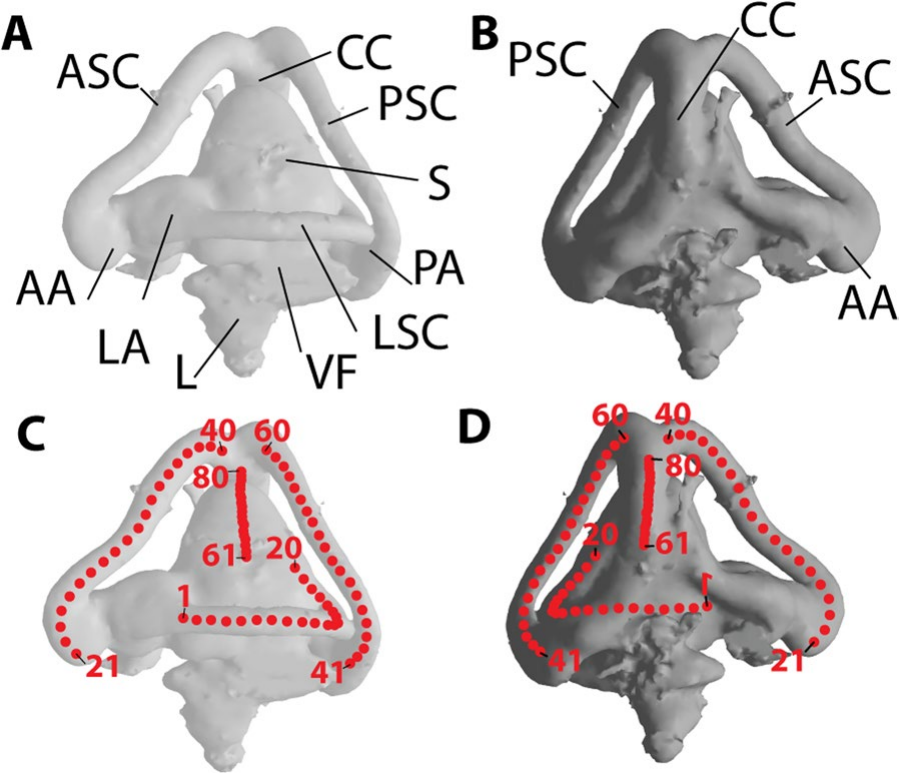
Landmarks on the endocast of the bony labyrinth of *Varanus niloticus* (PIMUZ A/III 0225). **A** Lateral view. **B** Medial view. **C** and **D** Same views as in A and B but with landmarks added. Each canal is described by 20 landmarks along a central streamline. *AA* anterior ampulla, *ASC* anterior semicircular canal, *CC* common crus, *L* lagena (including the cochlear duct), *LA* lateral ampulla, *LSC* lateral semicircular canal, *PA* posterior ampulla, *PSC* posterior semicircular canal, *S* sacculus, *VF* vestibular fontanelle

Midline streamlines (see [37]; a slightly different approach is followed in [11]) were landmarked to quantify the orientation and direction of each canal as opposed to surface landmarking, which can be affected by surface irregularities of each canal and low-resolution scans. Surface files (.ply or.stl) representing the semicircular canals of each specimen were imported into Meshtools [38] where the midline streamline endocasts of each canal were landmarked manually (Fig. 1) using an arbitrary number of points. The anterior and posterior canals were landmarked from the junction of their ampulla to the canal’s junction with the common crus. The horizontal canal was landmarked from the ampulla to the last visible portion of the canal on the sacculus. The common crus was landmarked from the lowest visible point on the sacculus to the junction with the anterior and posterior canals. These hand-placed points were then resampled into curves comprising twenty evenly spaced semilandmarks.

All subsequent analyses were performed in the R Statistical Environment v. 4.1 [39], using the packages *geomorph* v.4.0.1 [40], *caper* v.1.0.1 [41], and *ape* v.5.6-1 [42]; R functions used are listed as (*package: function*).

The 3D coordinates of the streamline semilandmarks were aligned using Procrustes superimposition and allowed to slide along tangents to the curves between the first and last point to minimize bending energy, centered around the origin, and scaled to same unit size (*geomorph: gpagen*). These points, the Procrustes residuals of the semicircular canal streamlines without the common crus, were used in the principal components analysis (PCA) (*geomorph: gm.prcomp*). The common crus was excluded because it was not uniformly visible in all taxa and because including it appears to introduce noise in the dataset (further explained in the Additional file 1).

### Body size

Three proxies for body size: body mass, snout-vent length (SVL; the length of the animal from the tip of the snout to the vent), and semicircular canal centroid size (the square-root of uncorrected distances of the semilandmark points from the centroid) were used, all Log-transformed and henceforth referred to only as body mass, SVL and centroid size. Because the sampled specimens do not have associated SVL measurements, we have used maximum SVL from the literature for each species. Because our specimens are not as long as the maximum recorded lengths for their species, we also use another indicator of individual size, centroid size. Centroid size captures an estimate of the actual size of the semicircular canals of a specimen as a proxy for head length and head size. Because body mass is correlated to inner ear size in mammals [43, 44], we test for a similar relationship in limbed squamates.

### Phylogenetic signal

**Fig. 2:**
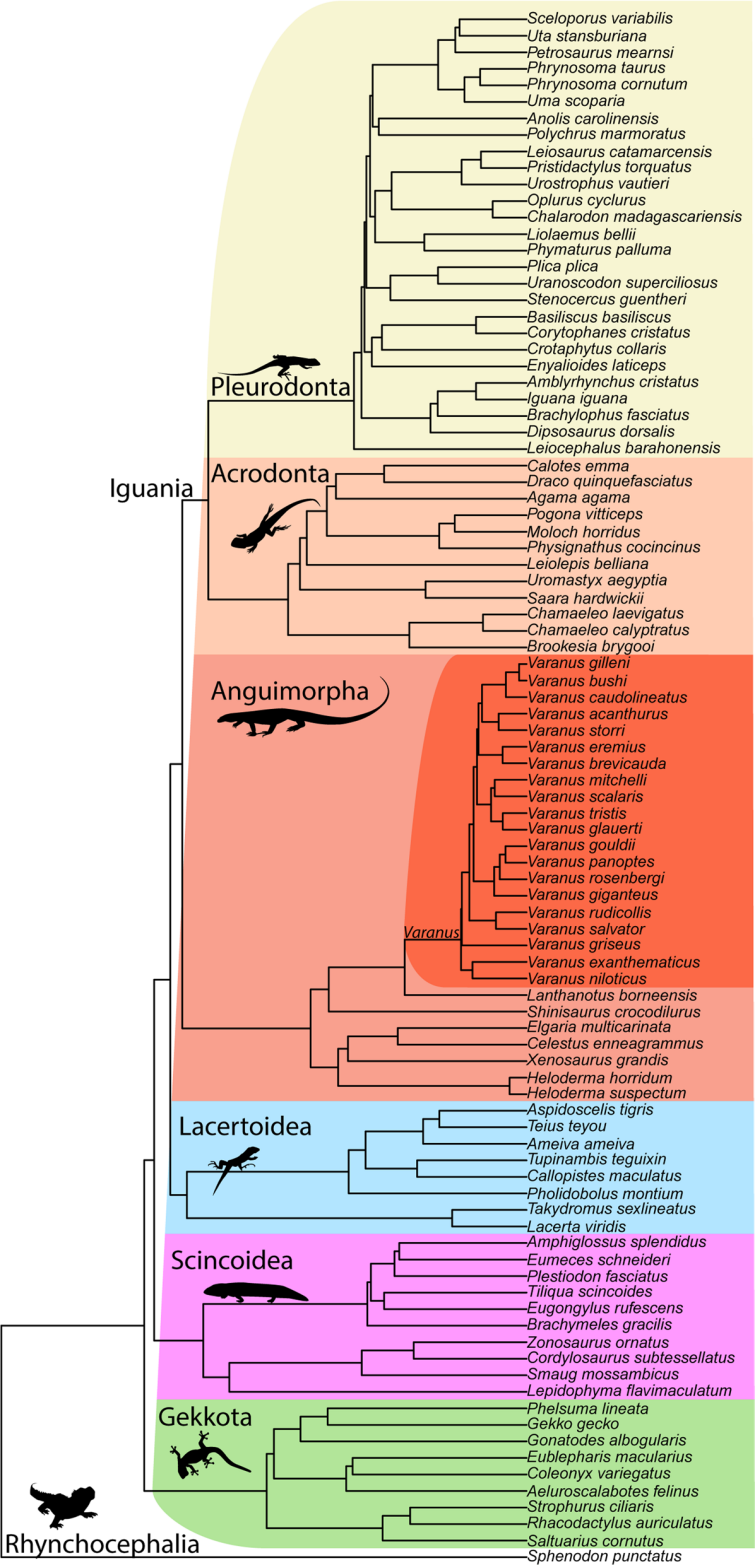
Phylogenetic tree of taxa used in comparative analyses. The tree is pruned from [45]. Colours correspond to the indicated taxonomic group in all figures

To account for species relationships in all statistical analyses, a time calibrated tree for squamate phylogeny [45] was pruned using *ape: drop.tip* [42] to the 94 species of squamates with legs and *Sphenodon* in this study (Fig. 2). This pruned tree was used to estimate phylogenetic influence in all analyses. All analyses were performed in the R Statistical Environment [39].

We tested phylogenetic signal in the shape of the semicircular canals among species. The amount of phylogenetic signal held within Procrustes residuals was estimated using the K_mult_ statistic (*geomorph: physignal*) [46], a multivariate version of K [47]. Like K, K_mult_ estimates the phylogenetic signal in a dataset assuming a Brownian motion model of evolution. Values near zero would be represented by a star tree where the variation is explained by things other than phylogeny, values closer to one suggest there is as much phylogenetic signal as would be expected under a Brownian motion model, and higher values are explained by more phylogenetic signal in the morphology than predicted under a Brownian motion model of evolution (see Additional file 1: Fig. S2).

### Integration

We assessed the degree of evolutionary integration (covariation) in shape among the three canals. To do this, the Procrustes residuals of the three semicircular canals (without the common crus) were examined using two-block partial least squares analysis with 1000 permutations in a phylogenetic framework (*geomorph: phylo.integration*) [48] to assess the null hypothesis that the canals are not integrated. Pairwise analysis of the three canals was performed.

### Life habit

To test for an association between lifestyle as proxy for locomotion and the morphology of the semicircular canals, the lifestyle habit of each species was recorded in four categories, using binary coding (yes or no): fossorial, arboreal, semiaquatic, terrestrial. Frequently, a taxon’s life habits cannot be described adequately by a single category, therefore taxa may have one, two or three life habits in combination; however, no taxon has all four life habits. For example, although marine iguanas *Amblyrhynchus cristatus* forage in water, they spend 95% of their time on land [49] to sun themselves on coastal rocks, compete for mating territories, and reproduce. Therefore, marine iguana are coded as terrestrial and semiaquatic. Not all semiaquatic taxa are terrestrial, for example the green iguana, *Iguana iguana* lives mainly in trees overhanging rivers, and if threatened, drops into the water to swim to safety [50, 51]. The green iguana, an adept swimmer, is coded here as arboreal and semiaquatic. It would be misleading to code the marine iguana as aquatic and the green iguana as arboreal when both taxa swim regularly. Taxa spending time in and on trees are classified as arboreal, and likewise those spending time on the ground are terrestrial, and taxa which are accomplished swimmers and spend time in water are semiaquatic. Those taxa which either stay for an extended time in burrows of their own creation or dig in leaf litter are classified as fossorial herein, understanding the limits to this category laid out previously [5]. Characterization of each species’ life habit comes from many sources (listed in the Additional file 1).

The correlation between semicircular canal shape (Procrustes residuals) and life habit was assessed using a phylogenetic least squares (PGLS) analysis for high dimensional data (*geomorph: procD.PGLS*) [52]. We fitted a PGLS on Procrustes residuals with all life habits fitted together and controlling for centroid size, body mass and SVL (Procrustes residuals ~ log centroid size + log body mass + log SVL + habits). To confirm the robustness of our results we fitted life habits independently controlling for the same measures (Procrustes residuals ~ centroid size + body mass + SVL + habit). In addition, to reveal the possible confounding effect of body size, we re-fitted the first model without correction for body mass and SVL (Procrustes residuals ~ log centroid size + habits). We used 1000 permutations for each model.

To assess the association of SVL, mass, and centroid size with a particular life habit, we performed a univariate PGLS (*caper: pgls*) with lambda transformation of branch length estimated by maximum likelihood. For all PGLS we used type III ANOVA to assess significance and estimate partial coefficients of determination for each predictor.

Results of Procrustes generalized least squares (PGLS) and comparison with habits are compiled into Tables 1 and 2.

**Table 1:** Results of phylogenetic generalized least squares (PGLS) with type III correction, giving coefficient of determination, F-statistic and P-values based upon 1000 permutations

	R^2^	F	Pr(> F)
a) shape ~ SVL + centroid size + body mass + terrestrial + arboreal + semiaquatic + fossorial			
Log SVL	0.01748	1.857	0.052947
Log centroid size	**0.02728**	**2.8985**	**0.001998**
Log body mass	0.01581	1.68	0.093906
Terrestrial	0.01429	1.5183	0.13986
Arboreal	**0.0225**	**2.3899**	**0.002997**
Semiaquatic	0.01454	1.545	0.110889
Fossorial	**0.02632**	**2.7956**	**0.003996**
b) shape ~ SVL + centroid size + body mass + habit (Single life habit at a time)			
Log SVL	0.0172	1.757	0.070929
Log centroid size	**0.02956**	**3.0194**	**0.000999**
Log body mass	0.01567	1.6005	0.10989
Terrestrial	0.01544	1.5772	0.128871
Arboreal	**0.02209**	**2.2735**	**0.00999**
Semiaquatic	0.01694	1.7327	0.067932
Fossorial	**0.0215**	**2.2106**	**0.025974**
c) shape ~ log centroid size + habit (Without the body size correction)			
Log centroid size	**0.05611**	**5.8057**	**0.000999**
Terrestrial	**0.02272**	**2.3510**	**0.017982**
Arboreal	**0.02204**	**2.2806**	**0.004995**
Semiaquatic	**0.02146**	**2.2203**	**0.015984**
Fossorial	**0.02557**	**2.6456**	**0.012987**

Bold values indicate significant correlations

**Table 2:** Phylogenetic generalized least squares comparison

	Estimate	Std. Error	Pr( >|t|)	R^2^
a) Centroid ~				
(Intercept)	2.382134	0.743793	0.001889	
Semiaquatic	**0.793951**	**0.176958**	**2.16E-05**	0.098
Fossorial	− 0.035202	0.271208	0.897019	0.021
Arboreal	− 0.064068	0.150843	0.672054	0.016
Terrestrial	− 0.309436	0.172557	0.076331	0.04
b) Body Mass ~				
(intercept)	2.420711	0.454778	7.58E-07	
Semiaquatic	**1.178979**	**0.220609**	**6.93E-07**	**0.16**
Fossorial	0.030608	0.289107	0.91592	0.000074
Arboreal	− 0.241607	0.185734	0.19668	0.015
Terrestrial	− 0.400515	0.238079	0.09602	0.025

Phylogenetic generalized least squares comparison of a) log-centroid size ~ and b) log-body mass ~. Bold values indicate significant correlations

### Allometry

We tested whether semicircular canal size (centroid size) predicts (in the statistical sense of correlated conditional to a model) SVL and body mass using a PGLS (type III, *geomorph: procD.pgls*) using log-transformed variables. Then we ran a multivariate regression using a PGLS to test for evolutionary allometry, the change in semicircular canal shape associated with evolutionary change in size (centroid size, SVL, and body mass) across a phylogeny [34]. Size variables were log-transformed and included together and evaluated with a Type III sums of squares approach because of their interaction. Maximum SVL from large sizes on record may correspond to SVL related allometry and it is a commonly available measurement. The null hypothesis of no correlation between size variation and shape variation along branches of the tree was evaluated through 1000 permutations. Visualizations of the shape variation predicted by size were done using a regression score approach [53].

## Results

### Integration

The shapes of the three canals are integrated with moderately strong covariation (mean of pairwise partial least squares correlations between canals; r-PLS = 0.729 Z = 8.22 P < 0.001), such that changes in shape of one canal are correlated with changes in shape in the others, and permutation analysis rejects the null hypothesis of no covariation. Therefore, all three semicircular canals are used together for subsequent analysis.

### Principal components and major axes of shape variation

**Fig. 3:**
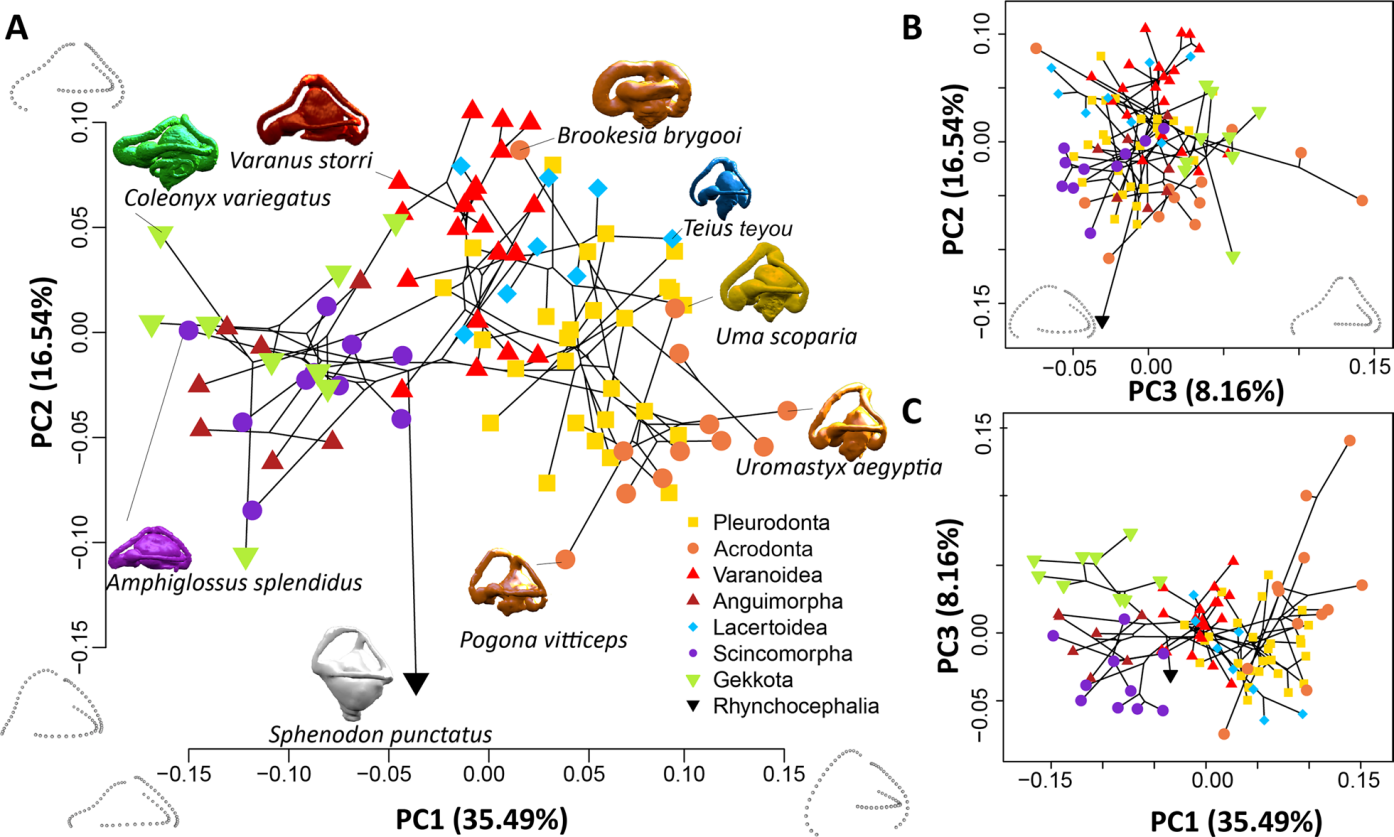
Morphospace of semicircular canal shape in 94 lepidosaur species. Taxa plotted on **A** PC1 and PC2, **B** PC3 and PC2, **C** PC1 and PC3 for the landmark coordinates of the three semicircular canals together, for each species, colored by clade. Phylogenetic tree from Fig. 2 is projected into morphospace to indicate the species relationships

The first three axes from the PCA account for 60.2% of the proportion of total variance (PC1: 35.492%, PC2: 16.543%, PC3: 8.163%). The closest to average shape (0,0) on PC1 and PC2 is *Phymaturus palluma*, a terrestrial pleurodont, nearby to both *Lacerta viridis* and *Varanus panoptes*. Each clade investigated here generally occupies a restricted portion of the morphospace on the principal components, but that space is not unique and overlaps with other clades (Fig. 3; see Additional file 1: Fig. S3). Gekkota, Scincomorpha, and Anguimorpha excepting *Varanus* overlap in the negative direction of PC1, whereas Lacertoidea, Acrodonta, Pleurodonta and *Varanus* occupy morphospace in the positive direction of PC1, and *Sphenodon* is in the middle. *Varanus* and Lacertoidea occupy positive values on PC2, Acrodonta are in the negative direction on PC2, and most species of the other clades are around the middle. *Sphenodon* falls furthest from the group on negative PC2.

**Fig. 4:**
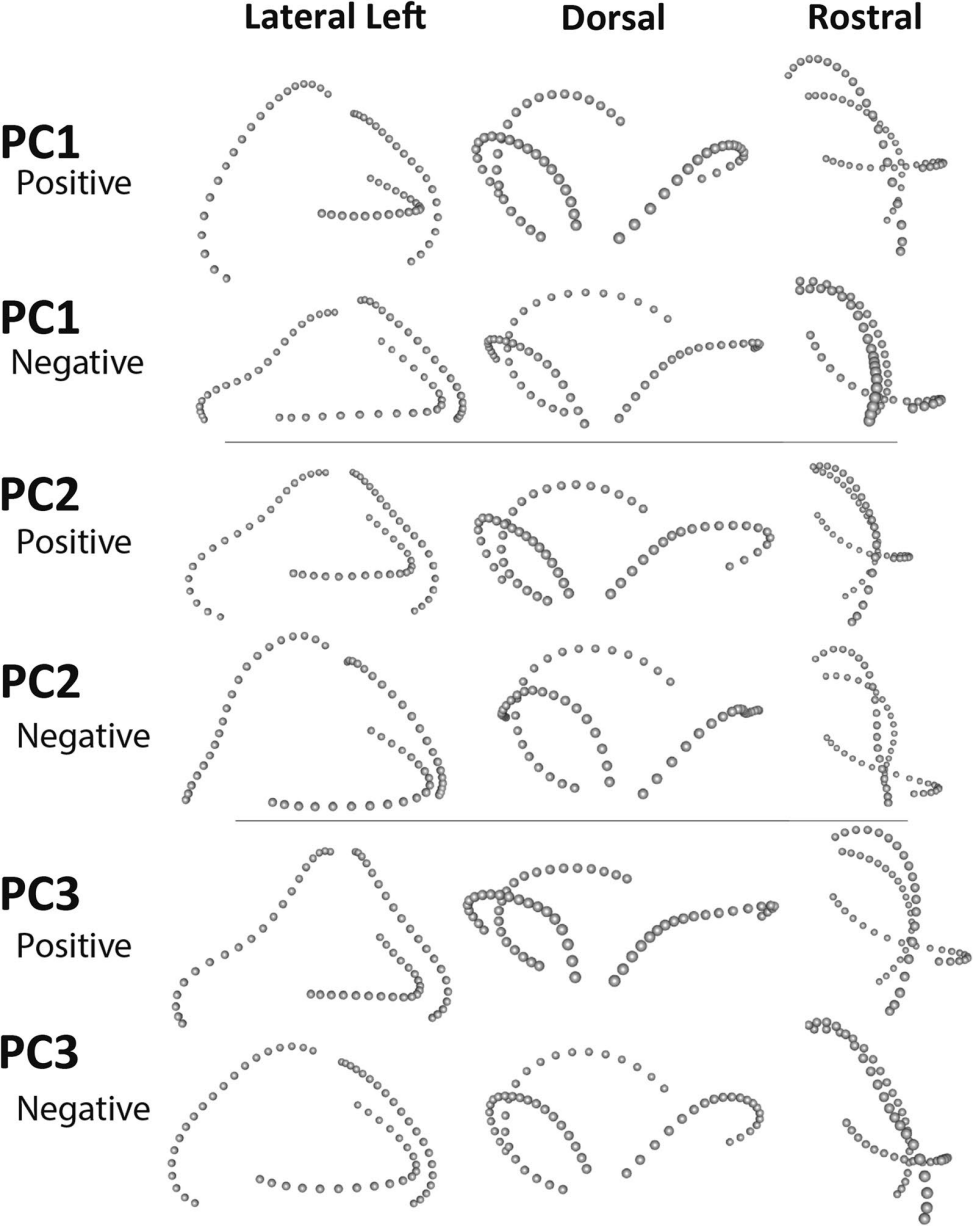
Shape configurations. These configurations depict shapes with maximum (positive) and minimum (negative) PC scores along the first three principal component axes, in three views

In general, each semicircular canal separates from a visible ampulla, curves to a point where it may or may not change direction, here called the inflection point but is sometimes absent, and continues, eventually joining either the common crus or into the body of the bony labyrinth. Deviations from this standard shape and the relative positions of the features described above are visible along the major principal components. Shape change of the semicircular canals along each PC axis away from the mean shape is best viewed in three dimensions (Fig. 4). On PC1, mean shape of the semicircular canals on the positive direction are more rounded, taller in the dorso-ventral direction, and the anterior and posterior semicircular canals separate from the ampulla ventral to the horizontal canal; whereas, in the negative direction the inflection point of the anterior canal increases and the canals elongate rostro-caudally and shorten dorso/ventrally. For increasingly negative values on PC2 the canals are more dorsal to the horizontal canal (exemplified by the rhynchocephalian *Sphenodon punctatus*), in the positive direction the connection to the ampulla of the anterior and posterior semicircular canals is more ventral to the horizontal canal. Shape change on the third principal component emphasizes the inflection point (concavity) in the anterior canal and the length of the horizontal canal in the positive direction, and roundness (convexity) of the anterior canal in the negative direction. PC3 also shows lateromedial compression and rostro-caudal extension of the semicircular canal system, in the positive direction which is best seen in dorsal view.

### Phylogenetic signal

There is significant phylogenetic signal in the morphology of the semicircular canals among limbed squamates (K_mult_ = 0.615, P < 0.001) (Additional file 1: Table S1). The value of K_mult_ is less than one, therefore the Procrustes residuals have phylogenetic signal but less than would be assumed under a Brownian motion model of evolution. Clades cluster in the morphospace defined by the first three principal components (Fig. 3). Centroid size also has significant phylogenetic signal (K = 0.453, P = 0.001) with a similarly low value of K indicating less phylogenetic signal than would be assumed under Brownian motion.

### Life habit

**Fig. 5:**
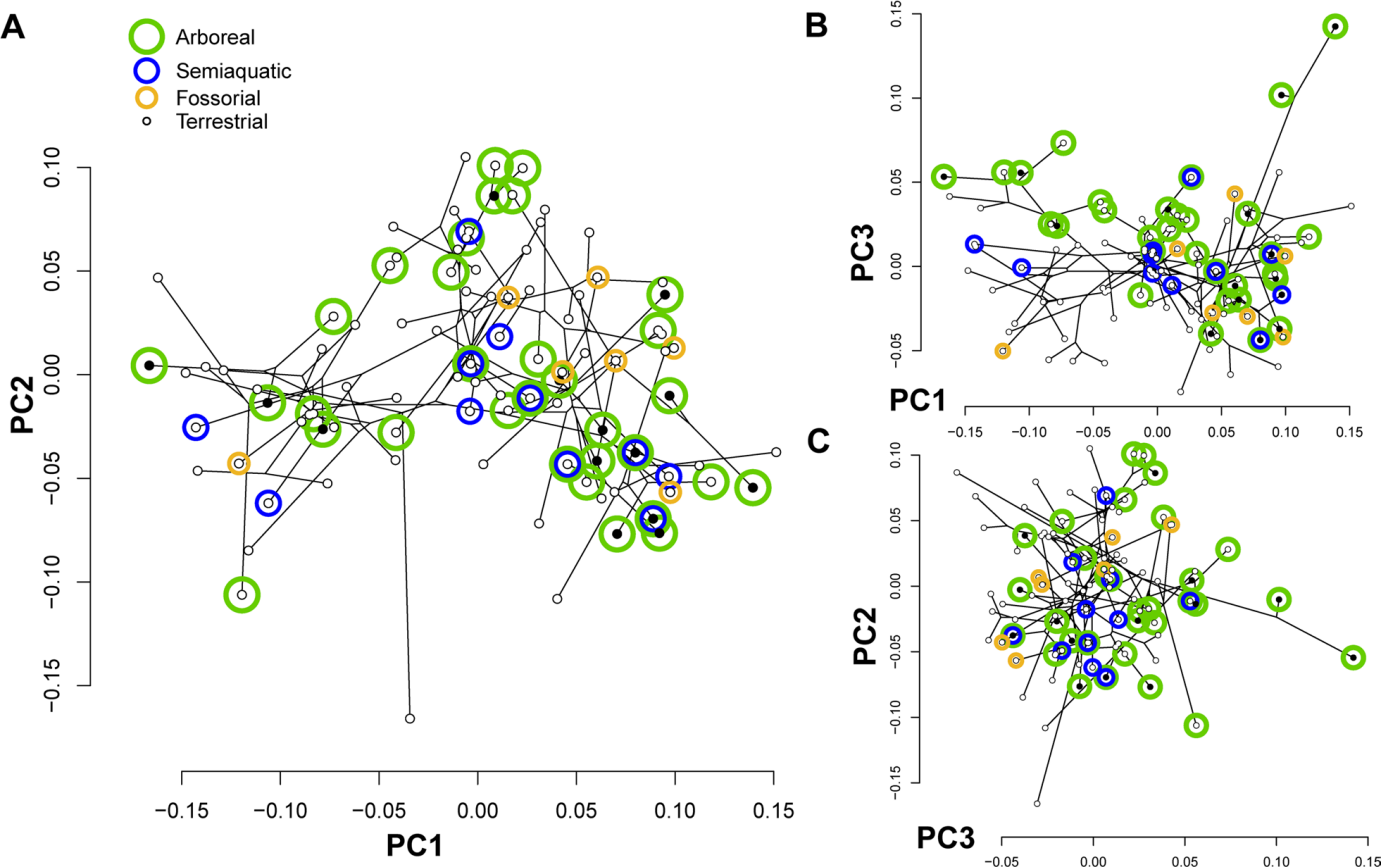
Life habits of each sampled species. The life habits are plotted on **A** PC1 and PC2, **B** PC1 and PC3, and **C** PC2 and PC3. Each species may have more than one life habit, and multiple life habits are plotted as concentric circles. Black circles are the central point for non-terrestrial species

When correcting for body size and centroid size, PGLS of the Procrustes residuals shows weak support for convergent canal shapes among life habit groups (Table 1a, b), and shape variation explained by life habit is low (R^2^ < 0.03), which is visually supported by the PCA morphospace and the lack of clustering of species in each group (Fig. 5). Arboreal and fossorial life habits are recovered as significant factors, albeit weakly predicting canal shape independently of which factors are used to control for body size (Table 1a–c). Terrestrial and semi aquatic life habits only predict canal shape when no correction for body size is used (Table 1c).

We would like to stress that if body size is not accounted for, spurious associations between shape and terrestrial or semiaquatic life habits are likely to be detected (Table 1c). Indeed, semiaquatic taxa tend to be larger (our largest taxa in the sample are all semiaquatic), and larger ears correspond to different shapes. Moreover, centroid size, body mass, and body length are not interchangeable proxies for body size. If only centroid size is used to correct for body size, and not body mass or SVL, then a component of shape is attributed to semiaquatic life habit.

### Allometry

**Fig. 6:**
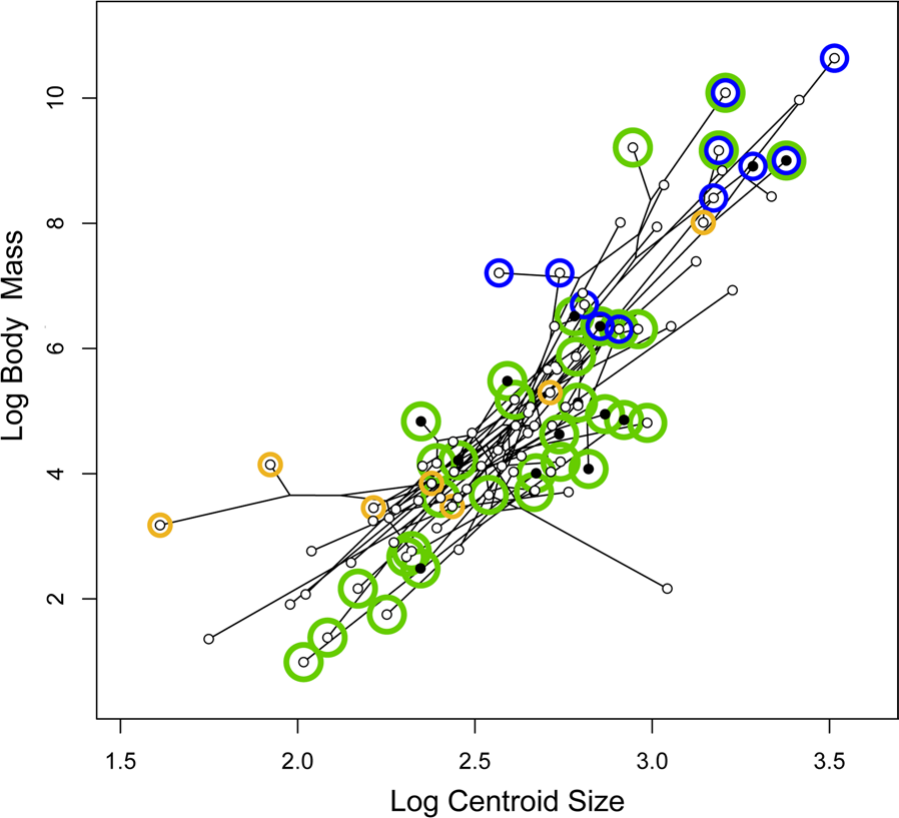
Relationship between centroid size and life habit mapped on species points as in Fig. 5

We find a small but significant allometric relationship between semicircular canal shape and centroid size (R^2^ = 0.0314 F_(1,92)_ = 3.185 P = 0.003), and SVL (R^2^ = 0.0186 F_(1,92)_ = 1.886 P = 0.043), but not with body mass (R^2^ = 0.156 F_(1,92)_ = 1.583 P = 0.111). Furthermore, PGLS revealed centroid size predicts body mass (R^2^ = 0.681 F_(1,92)_ = 196.76 P < 0.001; Fig. 6) and to a lesser extent SVL (R^2^ = 0.675 F_(1,92)_ = 191.13 P < 0.001) with moderate coefficients of determination.

## Discussion

### Life habits

Previous studies in different clades have suggested that aspects of inner ear morphology converge with ecology [5, 14, 17, 18, 24]. Our results from a sample of 94 species across squamates with legs do not support that hypothesis when controlling for the effects of phylogenetic relatedness and size.

### Life habit is not a strong predictor of semicircular canal shape

Within the category of fossorial taxa, other studies have recovered a specific type of inner ear morphology associated with fossorial taxa in amphibians [23, 24] and snakes [17]. That fossorial morphology is primarily characterized in amphibians and squamates by a notably large vestibule, increased ellipticity (described as anterior–posterior elongation), and is usually found among taxa with leg or eye reduction.

We do not examine the size or shape of the vestibule, sacculus, lagena, or canal thickness but can capture the relative lengths of the central streamlines. Although the sample includes few obligate fossorial taxa, and no legless head-diggers, we found fossoriality explains about 2% of the variation in shape of the inner ears of this broad group.

Fossorial taxa have specializations that might influence semicircular canal function in competing or complementary ways and disentangling these may prove fruitful. Three such specializations are reduced sight, leglessness, and head-digging. Reduced sight is common among fossorial taxa across phylogeny (amphisbaenians, skinks, moles, etc.) and reduces the need for gaze-stabilization. Comparing taxa with reduced sight from fossoriality, nocturnality, or because they dwell in a dark environment like the deep sea or caves would prove one testable measure. A second is size effects among small fossorial taxa. It is well described that the semicircular canals of smaller squamates have negative allometry and fill most of the posterior portion of the braincase [19] and therefore exhibit high ellipticity [33]. Finally, leglessness may change how taxa move, encouraging behaviors like head digging which subjects the skull to repetitive motion and significant strain. That strain may change skull morphology and thereby influence the shape of the semicircular canals. This could be contrasted between taxa that dig with limbs and their relatives that use head digging, and when size-corrected, comparing legless taxa with limbed relatives across clades with repeated limb-reduction, e.g., skinks, could provide the necessary contrast to answer this question. Disentangling the relative effects of morphologies (including canal thickness, e.g. [54] on fossorial mammals) and size associated with fossorial life would provide a step forward in understanding the way shape varies across the inner ears of limb-reduced and fossorial vertebrates.

Like fossorial taxa, arboreality explains 2% of the shape variation of the inner ears of taxa with that trait. Like fossorial life habit, evolution led to different solutions for living in an arboreal habitat. The effect of arboreality on the shape of the inner ear has been investigated in *Anolis* lizards [18] and snakes [5]. There are fewer drastic changes in body morphology associated with arboreal taxa than fossorial ones; those changes may be associated with microhabitats in the arboreal environment [18], or in the extreme case, near-flying locomotion like aerial descent with squirrels or squamates [15]. These changes are often solved in different and unique ways among arboreal vertebrates but using the same suite of features- specifically eye size, leg length, and head rotations.

### Body size, centroid size, and life habit

Including centroid size in models does not give the same result as accounting for body size and can lead to spurious associations if body size is not included, especially in aquatic taxa. Streamlines of semicircular canals as we used here, again, do not capture the thickness of canals, which may be an important indicator of some marine adaptions [55], but the general aspect ratio and shape is retained. Semicircular canal morphology associated with aquatic habits in reptiles has been described with flatter canals and a more antero-posteriorly (lower aspect ratio) expanded/dorsoventrally flattened aspect ratio [26]. These observations, however, were not assessed within a robust phylogenetic framework, with non-aquatic relatives, or with body size correction. As of yet further research is needed to predict semiaquatic life habit among squamates with legs.

### Phylogenetic signal of semicircular canal shape

Phylogenetic history contributes strongly to central streamline shape diversity among species (Fig. 2) and related species cluster in morphospace by clade. Some papers have analyzed semicircular canal shape without phylogenetic context where it is lacking [56] but it is clear that the papers that include it add to the confidence to their predictions [18, 55]. Furthermore, it appears that our broad clade investigation is less sensitive to life habit effects than smaller clades with high sample sizes [18]. While some features can be identified by eye without accounting for phylogenetic history, our results suggest phylogenetic history is an important driver of semicircular canal streamline shape across clades of lepidosaurs. Therefore, future studies to assess other predictors of semicircular canal streamline shape should control for phylogeny.

Some species have semicircular canal shape quite different from their closest relatives, and thus are positioned far from the other members of their clades along the principal components (Fig. 3). These outliers in the semicircular canal morphospace are taxa with unusual semicircular canals, and they have other unusual features. A notable outlier is a dwarf chameleon from Madagascar, *Brookesia brygooi*. Aside from being very small, this dwarf chameleon also has bulbous semicircular canals typical of dwarf chameleons [27], which are quite different from other chameleons. *Brookesia* have an otherwise unique method of tail-assisted walking, very slowly using its tail like an extra leg [27], locomotion quite unlike the actions of the much larger varanid lizards occupying that region of morphospace. Another outlier is *Phrynosoma*, which has the smallest semicircular canals of the taxa surveyed but is not the smallest species. It plots outside the normal allometric relationship of semicircular canal size and body mass of squamate reptiles, and is likely due to their shortened, highly ossified and ornamented posterior region of the skull. The inner ears of that clade may arrest development early, and there may be a unique functional aspect to the caudal region of the skull in these taxa. Third, the cat gecko, *Aeluroscalabotes felinus*, has a much lower PC2 value (the anterior and posterior semicircular canals do not extend as far below the horizontal canals, the aspect ratio of the landmarks is longer antero-posterior than they are dorso-ventrally) than other geckos and they notably do not have toe pads, instead using retractile claws and a prehensile tail to climb trees.

### Contributions from size and allometry

Allometry remains a small contributor to semicircular canal shape, and shape reflects variation in body mass but not independently of body length or centroid size. Semicircular canal size (centroid size) predicts body size (SVL and mass), therefore larger squamates generally have larger semicircular canals (Fig. 6).

We also find the previously documented correlation between size and life habit [35] that semiaquatic squamates are relatively larger. The largest species in our dataset is semiaquatic, the varanid *Varanus salvator*, the Asian Water monitor, among other semiaquatic varanids (Figs. 3, 5, 6). Semiaquatic taxa have a greater mass than their SVL would predict, likely aiding in heat retention while swimming [35], or possibly because semiaquatic squamates have disproportionately heavily muscled tails for swimming.

Allometry may be more meaningful for smaller taxa where small lizards solve the reduction in sensitivity in the semicircular canals from a flatter skull by increasing the ellipticity of the canals to fill a larger space [33]. Although ellipticity reduces sensitivity in the semicircular canals, for a given centroid size, having large elliptical semicircular canals improves sensitivity over small circular semicircular canals. This could also contribute to the strange shape of the semicircular canals in the miniature chameleon *Brookesia brygooi.* The constraints on semicircular canal shape from the size of the taxon in question can be considered when discussing shapes and comparison across taxa where some species are very small.

## Conclusions

Variation in shape in semicircular canals among limbed lepidosaurs is explained by phylogenetic history, therefore semicircular canal shape could be a predictor of phylogeny. On the other hand, shape is likely a poor predictor of life habit on large-clade scales. Predicting life habit from semicircular canal shape is likely more powerful in restricted clades of squamates [5, 18]. Confidently inferring the life habits of extinct taxa without a well sampled set of known close relatives seems unlikely. We found shape covaries with fossorial and arboreal life habits. Terrestrial or semiaquatic life habits covary with shape only when body size is not corrected for. The relationship between centroid size and body mass could also be used to estimate body mass and SVL in extinct squamates where only a skull is known (Additional file 2**, **Additional file 3).

While predicting life habit of distantly related taxa from the shape of semicircular canals measured by central streamlines is unlikely, we show there is hope for using central streamlines as another line of evidence to infer the phylogenetic placement of taxa, using centroid size to estimate body mass, and to maybe highlight species with heretofore unknown morphological peculiarities by observing inner ears that are much different from their close relatives.

## Supplementary Information

**Additional file 1.** Additional information on the life habit, inclusion tests, and sampled inner ear models.

**Additional file 2.** Table compiling specimen data.

**Additional file 3. **Curve landmark data used in the manuscript.

## Data Availability

All the data are included in the present study and accompanying additional information. The models of the inner ears and selected x-ray micro computed tomographs scans (see Materials and Methods) are made available on Morphosource (https://www.morphosource.org/) under repository project ID: 000508406. The R data have been uploaded on Figshare and can be accessed via the following link: https://figshare.com/s/0a4a6a118fa4b91aab59 [https://doi.org/10.25909/19785148].
