# Supplementary material for: Semicircular canal shape diversity among modern lepidosaurs: life habit, size, allometry
Source: BMC Ecol Evol. 2023 Apr 12;23:10. doi: 10.1186/s12862-023-02113-1 (PMC10091843; doi:10.1186/s12862-023-02113-1)
Supplement: Supplementary file 1 — Additional file 1. Additional information on the life habit, inclusion tests, and sampled inner ear models. [file 12862_2023_2113_MOESM1_ESM.docx]

Supplemental Material for

“**Semicircular canal shape diversity among modern lepidosaurs: Life habit, size, allometry**”

Ashley E. Latimer, Emma Sherratt, Timothée Bonnet, Torsten M. Scheyer

**Habit**

Habit categories for each taxon are based on literature cited in the supplementary materials (see supplementary Excel file) and expanded here [1-45].

**Inclusion tests**

To test if sliding semilandmarks should be included in the analysis, and if the common crus brings information or noise, generalized Procrustes analyses (*package:geomorph*, *function: gpagen*) and phylogenetic signal (*geomorph:physignal*) were implemented.

**Common Crus:** The common crus vary in visibility by clade, and their extent is poorly defined (Supplementary Fig. S1). The proportion of variance from each of the three principal components for analyses with the common crus were lower than without them, and the amount of phylogenetic signal was likewise lower (Supplementary Figs. S1-S3; Supplementary Table S1). Therefore, including the common crus increases the amount of noise in the dataset, and was excluded.

**Sliding semilandmarks:** The sliding semilandmarks were developed for two dimensional (2D) landmarking to reduce the amount of artifact from evenly spaced space landmarks on 2D surface [46]. We tested this in three cases, without sliders, with curve sliders using bending energy, and third, curve sliders using procrustes distances. They are evaluated by looking at the recovered phylogenetic signal, and we assume that a larger phylogenetic signal would not be recovered by chance.

Bending energy recovers a greater phylogenetic signal than no sliders when the common crus is excluded and included (Supplementary Fig. S2). Procrustes distances, surprisingly are worse than excluding sliders when the common crus is excluded, but performed better than bending energy when it the common crus was included. The overall higher phylogenetic signal recovered by sliding the semilandmarks with bending energy and the unpredictable performance of Procrustes distances show bending energy is the better choice for semilandmarks sliding here. The curious performance of procrustes distances here suggests it may be better to use unslid data than the Procrustes distances, but if bending energy is an option it should be used instead.


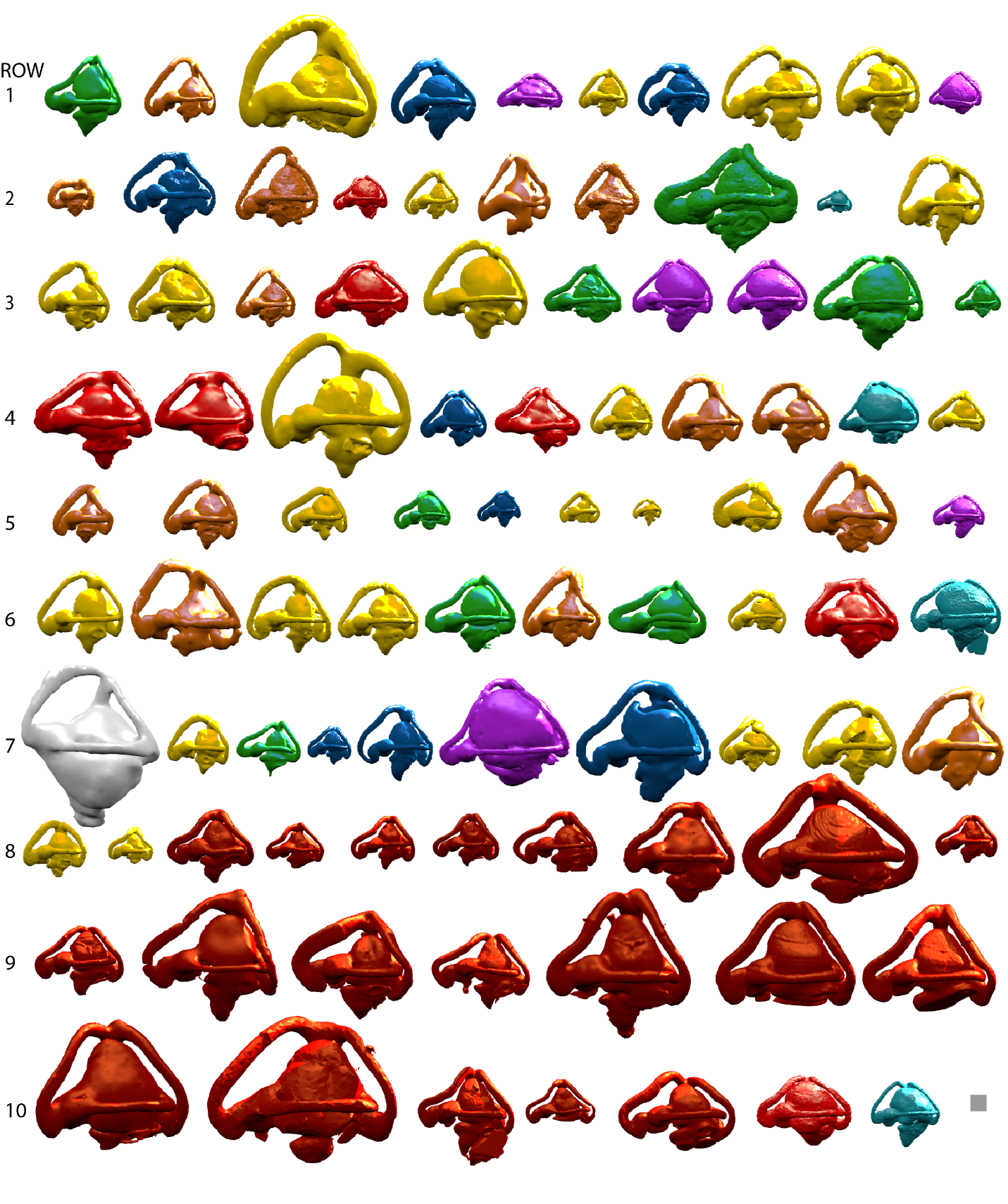


**Supplemental Fig. S1.** Inner ears of all specimens to scale, box is 1mm square, in alphabetical order with row numbers. Taxon numbers below correspond to the principal components in the Supplemental Fig. S3. Colors correspond to the taxon colors; Gekkota green, Scincoidea purple, Lacertoidea blue, Anguimorpha red, Acrodonta orange, Pleurodonta yellow, *Sphenodon* white. **ROW 1:** 1 *Aeluroscalabotes* *felinus*, 2 *Agama* *agama*, 3 *Amblyrhynchus* *cristatus*, 4 *Ameiva* *ameiva*, 5 *Amphiglossus* *splendidus*, 6 *Anolis* *carolinensis*, 7 *Aspidoscelis* *tigris*, 8 *Basiliscus* *basiliscus*, 9 *Brachylophus* *fasciatus*, 10 *Brachymeles* *gracilis****.***

**ROW 2:** 11 *Brookesia* *brygooi*, 12 *Callopistes* *maculatus*, 13 *Calotes* *emma*, 14 *Celestus* *enneagrammus*, 15 *Chalarodon* *madagascariensis*, 16 *Chamaeleo* *calyptratus*, 17 *Chamaeleo* *laevigatus*, 18 *Coleonyx* *variegatus*, 19 *Cordylosaurus* *subtessellatus*, 20 *Corytophanes* *cristatus*.

**ROW3:** 21 *Crotaphytus* *collaris*, 22 *Dipsosaurus* *dorsalis*, 23 *Draco* *quinquefasciatus*, 24 *Elgaria* *multicarinata*, 25 *Enyalioides* *laticeps*, 26 *Eublepharis* *macularius*, 27 *Eugongylus* *rufescens*, 28 *Eumeces* *schneideri*, 29 *Gekko* *gecko,* 30 *Gonatodes* *albogularis.*

**ROW 4:** 31 *Heloderma* *horridum*, 32 *Heloderma* *suspectum*, 33 *Iguana* *iguana*, 34 *Lacerta* *viridis*, 35 *Lanthanotus* *borneensis*, 36 *Leiocephalus* *barahonensis*, 37 *Leiolepis* *belliana*, 38 *Leiosaurus* *catamarcensis*, 39 *Lepidophyma* *flavimaculatum,* 40 *Liolaemus* *bellii*.

**ROW 5:** 41 *Moloch* *horridus*, 42 *Oplurus* *cyclurus*, 43 *Petrosaurus* *mearnsi*, 44 *Phelsuma* *lineata*, 45 *Pholidobolus* *montium*, 46 *Phrynosoma* *cornutum*, 47 *Phrynosoma* *taurus*, 48 *Phymaturus* *palluma*, 49 *Physignathus* *cocincinus,* 50 *Plestiodon* *fasciatus*.

**ROW 6:** 51 *Plica* *plica*, 52 *Pogona* *vitticeps*, 53 *Polychrus* *marmoratus*, 54 *Pristidactylus* *torquatus*, 55 *Rhacodactylus* *auriculatus*, 56 *Saara* *hardwickii*, 57 *Saltuarius* *cornutus*, 58 *Sceloporus* *variabilis*, 59 *Shinisaurus* *crocodilurus,* 60 *Smaug* *mossambicus.*

**ROW 7:** 61 *Sphenodon* *punctatus*, 62 *Stenocercus* *guentheri*, 63 *Strophurus* *ciliaris*, 64 *Takydromus* *sexlineatus*, 65 *Teius* *teyou*, 66 *Tiliqua* *scincoides*, 67 *Tupinambis* *teguixin*, 68 *Uma* *scoparia*, 69 *Uranoscodon* *superciliosus*, 70 *Uromastyx* *aegyptia*.

**ROW 8:** 71 *Urostrophus* *vautieri*, 72 *Uta* *stansburiana*, 73 *Varanus* *acanthurus*, 74 *Varanus* *brevicauda*, 75 *Varanus* *bushi*, 76 *Varanus* *caudolineatus*, 77 *Varanus* *eremius*, 78 *Varanus* *exanthematicus*, 79 *Varanus* *giganteus*, 80 *Varanus* *gilleni*.

**ROW 9:** 81 *Varanus* *glauerti*, 82 *Varanus* *gouldii*, 83 *Varanus* *griseus*, 84 *Varanus* *mitchelli*, 85 *Varanus* *niloticus*, 86 *Varanus* *panoptes*, 87 *Varanus* *rosenbergi*.

**ROW 10:** 88 *Varanus* *rudicollis*, 89 *Varanus* *salvator*, 90 *Varanus* *scalaris*, 91 *Varanus* *storri*, 92 *Varanus* *tristis*, 93 *Xenosaurus* *grandis*, 94 *Zonosaurus* *ornatus.*


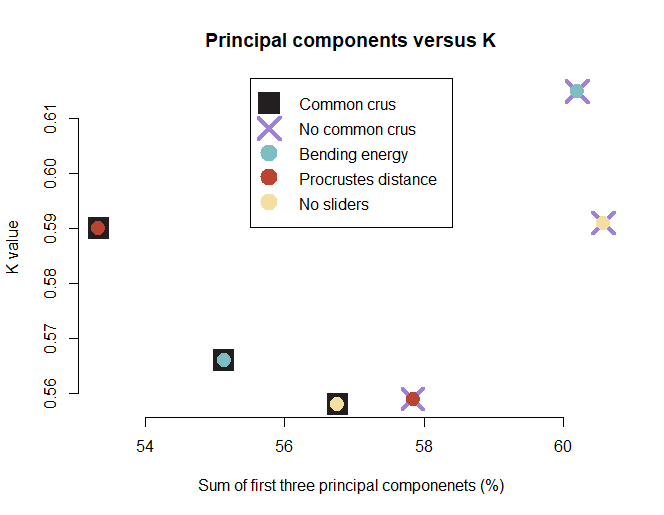


**Supplemental Fig. S2.** Graph of the different outputs for the aligned coordinates under different conditions, phylogenetic signal (K) indicates phylogenetic influence.


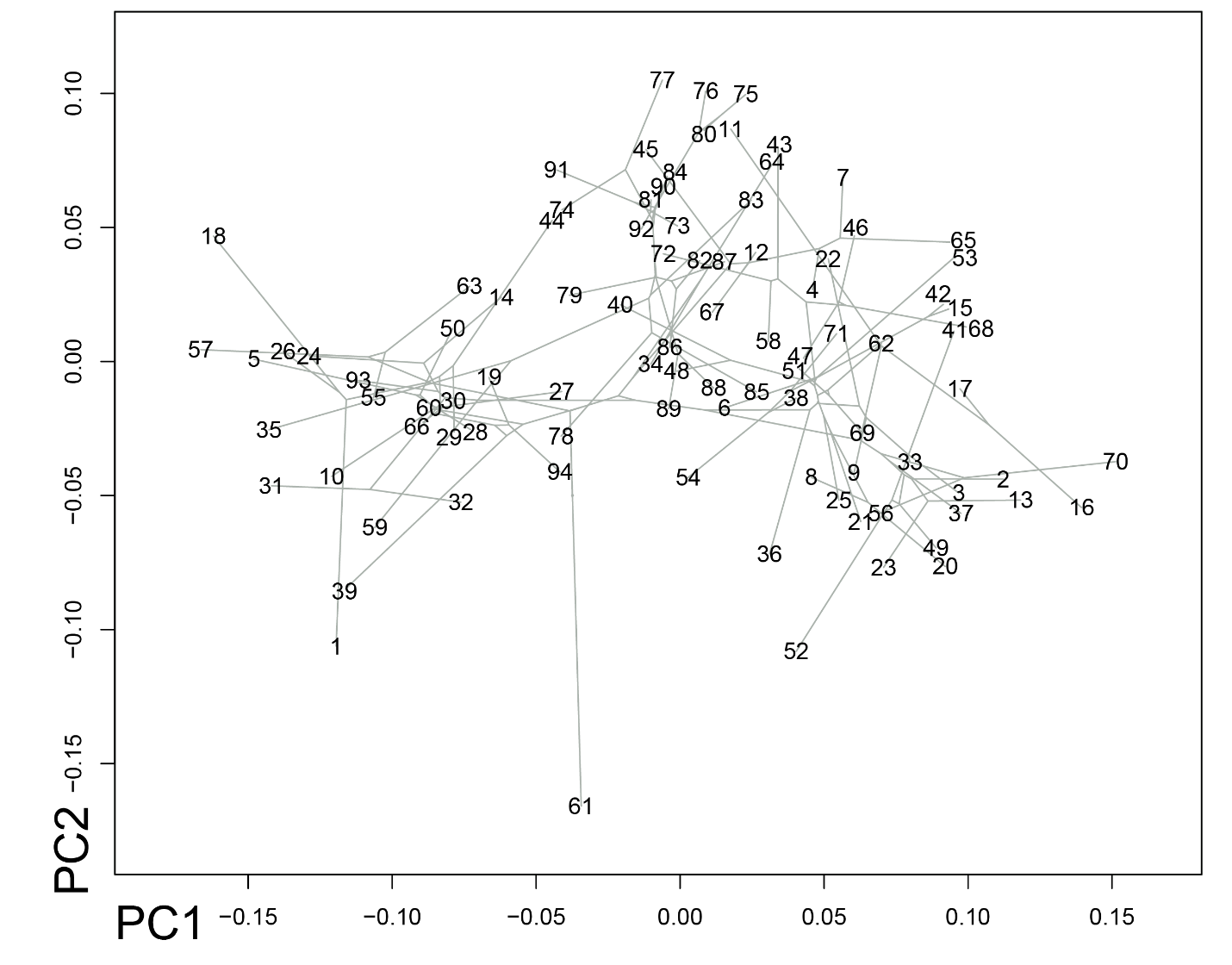


**Supplemental Fig. S3.** Principal components 1 and 2, taxon numbers are alphabetical and correspond to the caption of Supplemental Fig. 1.

**Supplemental Table 1**. The phylogenetic signal and proportion of variance in each of the three principal component axes with and without the common crus, bending energy (BE), or Procrustes distances (PD). K is the phylogenetic signal in the data [47].

| **Common Crus** | **Sliders** | **K** | **PC1** | **PC2** | **PC3** | **Sum of PC1-3** |
| --- | --- | --- | --- | --- | --- | --- |
|  | BE | 0.607 | 33.59 | 15.56 | 8.86 | 58.008 |
| CC | BE | 0.559 | 26.98 | 16.91 | 9.12 | 53.01 |
|  |  | 0.557 | 28.71 | 21.49 | 9.06 | 59.26 |
| CC |  | 0.549 | 24.31 | 21.16 | 9.1 | 54.568 |
|  | PD | 0.554 | 26.78 | 20.14 | 8.91 | 55.82 |
| CC | PD | 0.583 | 25.51 | 17.85 | 8.68 | 52.039 |

**References**

1. Zaaf A, Van Damme R: **Limb proportions in climbing and ground-dwelling geckos (Lepidosauria, Gekkonidae): A phylogenetically informed analysis**. *Zoomorphology* 2001, **121**:45–53.

2. Das I: **Lizards of Borneo: A Pocket Guide**. Kota Kinabalu: *Natural History Publications (Borneo),* 2004.

3. Yeboah S: **Observations on territory of the rainbow lizard, *Agama agama***. *African Journal of Ecology* 1982, **20**:187–192.

4. Wiens JJ, Brandley MC, Reeder TW: **Why does a trait evolve multiple times within a clade? Repeated evolution of snakelike body form in squamate reptiles**. *Evolution* 2006, **60**:123–141.

5. La Marca E, Soriano P: **Reptiles de los Andes de Venezuela**. Mijares A, Esqueda LF, Perez JEG, Navarrete LF, editors. Merida: BIOGEOS; 2004.

6. Greenberg N, Crews D: **Endocrine and behavioral responses to aggression and social dominance in the green anole lizard, *Anolis carolinensis***. *General and Comparative Endocrinology* 1990, **77**:246–255.

7. Mitchell JC: **Ecology of southeastern Arizona whiptail lizards (*Cnemidophorus*: Teiidae): population densities, resource partitioning, and niche overlap**. *Canadian Journal of Zoology* 1979, **57**:1487–1499.

8. Ord TJ, Blumstein DT, Evans CS: B**cology and signal evolution in lizards.** *Biological Journal of the Linnean Society* 2002, **77**:127–148.

9. Tolley KA, Herrel A (editors): **The Biology of Chameleons**. Berkeley: University of California Press, 2014.

10. Fuentes ER: **Ecological convergence of lizard communities in Chile and California.** *Ecological Society of America,* 1976, **57**:3–17.

11. Esquerré D, Núñez H: **Reptiles de la Región Metropolitana de Chile. 1^st^ Ed.** Pedreros AM, editor. Valdiva: CEA Ediciones, 2017.

12. Campbell JA, Camarillo R JL: **A new lizard of the genus *Diploglossus* (Anguidae: Diploglossinae) from Mexico, with a review of the Mexican and Northern Central American species**. *Herpetologica* 1994, **50**:193–209.

13. Higham TE, Jayne BC: **Locomotion of lizards on inclines and perches: hindlimb kinematics of an arboreal specialist and a terrestrial generalist**. *Journal of Experimental Biology* 2004, **207**:233–248.

14. Grismer LL: **Amphibians and Reptiles of Baja California.** Berkeley: University of California Press, 2002.

15. Branch B: **A Photographic Guide to Snakes and Other Reptiles of Southern Africa.** Cape Town: Struik Publishers, 2002.

16. Badger D, Netherton J: **Lizards: A Natural History of Some Uncommon Creatures - Extraordinary Chameleons, Iguanas, Geckos, & More.** Cornell K, editor. St. Paul: Voyageur Press, 2006.

17. Irschick DJ, Jayne BC: **Comparative three-dimensional kinematics of the hindlimb for high-speed bipedal and quadrupedal locomotion of lizards**. *Journal of Experimental Biology* 1999, **202**:1047–1065.

18. Duellman WE: **The Lives of Amphibians and Reptiles in an Amazon Rainforest**. Bauer AM, editor. Ithica: Comstock Publishing Associates, 2005.

19. Cogger H: **Reptiles and Amphibians of Australia. 7^th^ Ed.** Collingswood: Csiro Publishing, 2014.

20. McCoy M: **Reptiles of the Solomon Islands.** Sofia: Pensoft Publishers, 2006.

21. Miranda JP, Ricci-Lobão A, Rocha CFD: **Influence of structural habitat use on the thermal ecology of *Gonatodes humeralis* (Squamata: Gekkonidae) from a transitional forest in Maranhão, Brazil**. *Zoologia (Curitiba)* 2010, **27**:35–39.

22. Tulli MJ, Cruz FB, Herrel A, Vanhooydonck B, Abdala V: **The interplay between claw morphology and microhabitat use in neotropical iguanian lizards.** *Zoology* 2009, **112**:379–392.

23. Glaw F, Vences M: **Field Guide to the Amphibians and Reptiles of Madagascar. 3^rd^ Ed.** Frosch Verlag, 2007.

24. Adolphs K, Bates MF: ***Cordylosaurus subtessellatus*.** *IUCN Red List of Threatened Species* 2010, e.T178341A7526963 [accessed and cited 2018 Jan 25]. Available from: http://dx.doi.org/10.2305/IUCN.UK.2010-4.RLTS.T178341A7526963.en.

25. Davis DD: **Behavior of the lizard *Corythophanes cristatus***. *Fieldiana Zoology* 1953, **35**:1–8.

26. Sunyer J, Chaves G, Porras LW, Lamar W, Solórzano A: ***Lepidophyma flavimaculatum***. *IUCN Red List of Threatened Species* 2013, e.T197495A2490538 [accessed and cited 2018 Jan 25]. Available from: http://dx.doi.org/10.2305/IUCN.UK.2013-2.RLTS.T197495A2490538.en.

27. Nunez H. EP, Mella J: ***Liolaemus bellii***. *IUCN Red List of Threatened Species* 2016 e.T56051706A56051708. 2016 [accessed and cited 2018 Jan 25]. Available from: http://dx.doi.org/10.2305/IUCN.UK.2016-1.RLTS.T56051706A56051708.en.

28. Vences M: ***Phelsuma*** ***lineata***. *IUCN Red List of Threatened Species* 2011 e.T172826A6925369 [accessed and cited 2018 Jan 25]. Available from: http://dx.doi.org/10.2305/IUCN.UK.2011-2.RLTS.T172826A6925369.en.

29. Münchenberg T, Wollenberg KC, Glaw F, Vences M: **Molecular phylogeny and geographic variation of Malagasy iguanas (*Oplurus* and *Chalarodon*)**. *Amphibia-Reptilia* 2008, **29**:319–27.

30. Tikader BK, Sharma RC: **Handbook of Indian Lizards.** Calcutta: Zoological Survey of India, 1992.

31. Blanc CP, Carpenter CC: **Studies on the Iguanidae of Madagascar III. Social and reproductive behavior of *Chalarodon madagascariensis***. *Journal of Herpetology* **1969**: 125–134.

32. Vitt L, Magnusson W, Pires TCA, Lima AP. Guide to the lizards of Reserva Adolpho Ducke. Manaus: Áttema Design Editorial; 2008.

33. de Lisle HF. Behavioral ecology of the banded rock lizard (*Petrosaurus mearnsi*). Bull. South. Calif. Acad. Sci. 1991;90:102–17.

34. Breckenridge WJ: **Reptiles and Amphibians of Minnesota**. Minneapolis: The University of Minnesota Press, 1944.

35. Swan M, Watharow S: **Snakes , Lizards and Frogs**. Collingwood: Csiro Publishing, 2005.

36. Chester SR. **A Wildlife Guide to Chile: Continental Chile, Chilean Antarctica, Easter Island, Juan Fernández Archipelago. 1^st^ Ed.** Princeton: Princeton University Press, 2008.

37. Laspiur A, Acosta JC, Abdala CS: **A new species of *Leiosaurus* (Iguania: Leiosauridae) from central-western Argentina**. *Zootaxa* 2007, **1470**:47–57.

38. Bauer AM, Sadlier RA: **The Herpetofauna of New Caledonia**. Ithaca: Society for the Study of Amphibians and Reptiles, in cooperation with the Institut de Recherche pour le Développement; 2000.

39. Savage JM: **The Amphibians and Reptiles of Costa Rica: A Herpetofauna between Two Continents, between Two Seas**. Chicago: University of Chicago Press, 2002.

40. Nguyen TQ, Hamilton P, Ziegler T. ***Shinisaurus crocodilurus***. *IUCN Red List Threat. Species* 2014 e.T57287221A57287235 [accessed and cited 2017 Aug 18]. Available from: http://dx.doi.org/10.2305/IUCN.UK.2014-1.RLTS.T57287221A57287235.en.

41. Pianka ER, Pianka HD: **Comparative ecology of twelve species of nocturnal lizards (Gekkonidae) in the Western Australian desert.** *Copeia* 1976, **1976(1)**:125–142.

42. Auliya M: ***Takydromus sexlineatus***. *IUCN Red List of Threatened Species* [accessed and cited 2018 Jan 25] 2010 e.T178424A7544274. 2010. Available from: http://dx.doi.org/10.2305/IUCN.UK.2010-4.RLTS.T178424A7544274.en.

43. Wilms T, Eid EKA, Al Johany AMH, Amr ZSS, Els J, Baha El Din S, Disi AM., Sharifi M, Papenfuss T, Shafiei Bafti S, Werner YL: ***Uromastyx aegyptia*** (errata version published in 2017). *IUCN Red List of Threatened Species* 2012 e.T164729A115304711. 2017 [cited 2017 Aug 18]. Available from: http://dx.doi.org/10.2305/IUCN.UK.2012.RLTS.T164729A1071308.en

44. Köhler G: **Reptiles of Central America**. Offenbach: Herpeton Verlag, 2003.

45. Winton WM. **Habits and behavior of the Texas horned lizard, *Phrynosoma cornutum*, Harlan**. I. Copeia. 1916;36:81–84.

46. Perez SI, Bernal V, Gonzalez PN: **Differences between sliding semi-landmark methods in geometric morphometrics, with an application to human craniofacial and dental variation**. *Journal of Anatomy* 2006, **208**:769–784.

47. Adams DC: **A generalized K statistic for estimating phylogenetic signal from shape and other high-dimensional multivariate data**. *Systematic Biology* 2014, **63**:685–697.
